# Impact of hyperbaric oxygen as initial treatment on idiopathic sudden sensorineural hearing loss prognosis: a propensity scores matching analysis

**DOI:** 10.1016/j.bjorl.2026.101775

**Published:** 2026-05-17

**Authors:** Sanlin Xie, Weihong Hong, Lipeng Huang, Yongjun Hong

**Affiliations:** a Xiamen University, School of Medicine, Zhongshan Hospital of Xiamen University, Department of Otolaryngology Head and Neck Surgery, Xiamen, China; b Xiamen University, School of Medicine, Xiang’an Hospital of Xiamen University, Department of Thoracic, Xiamen, China

**Keywords:** Idiopathic sudden sensorineural hearing loss, Hyperbaric oxygen therapy, Prognosis, Propensity score matching

## Abstract

**Objective:**

This study aimed to evaluate the effect of Hyperbaric Oxygen Therapy (HBOT) combined with steroids as initial treatment on the prognosis of patients with Idiopathic Sudden Sensorineural Hearing Loss (ISSNHL) by using propensity score matching (PSM) to balance covariates between groups.

**Methods:**

A case-control study design was adopted, including 413 ISSNHL patients admitted to our center from January 2021 to June 2024. Based on treatment protocols, patients were divided into two groups: the therapy group received steroids combined with HBOT (intravenous + intratympanic glucocorticoids + HBOT), while the control group received steroids alone (intravenous + intratympanic glucocorticoids). Audiometric evaluation was performed using pure-tone audiometry. PSM was applied in a 1:1 ratio, yielding 135 matched patients in each group. Clinical characteristics and treatment outcomes were then assessed.

**Results:**

Before PSM, significant differences were observed between the therapy and control groups in baseline hearing thresholds, severity of hearing loss, and hearing curve classification. After PSM, all imbalanced covariates were effectively balanced. Prior to matching, the overall hearing recovery rate significantly differed between the two groups (p = 0.02). However, after PSM, no statistically significant difference was found in the overall hearing recovery rate (p = 0.180).

**Conclusion:**

The addition of Hyperbaric Oxygen Therapy (HBOT) as an initial treatment modality for ISSNHL, when combined with intravenous and intratympanic steroid therapy, does not provide additional improvement in outcomes for patients.

**Level of evidence:**

3.

## Highlights

• To assess the impact of HBOT as an initial treatment on ISSNHL prognosis.
• HBOT combined with steroids did not significantly improve hearing recovery compared to steroids alone after PSM.
• Propensity Score Matching (PSM) was used to balance covariates between groups and reduce confounding factors.

## Introduction

Idiopathic Sudden Sensorineural Hearing Loss (ISSNHL) is an acute hearing loss of unknown origin, characterized by a rapid decline in hearing to its lowest level within 72 h. Pure-tone audiometry shows a hearing loss of ≥30 dB in at least three adjacent frequencies.^1^ According to statistics, it is estimated that by 2030, sudden deafness will rise from the 14th to the 7th leading cause of global disease burden.^2^ The exact etiology of ISSNHL remains unclear, but several potential causes have been proposed, including viral infections, microcirculatory disorders of the inner ear, and autoimmune diseases.^3^ Currently, a range of treatment modalities are available for ISSNHL, with Intravenous Steroid (IVS) and Intra-Tympanic (IT) steroid injections representing the cornerstone of first-line therapy. Hyperbaric Oxygen Therapy (HBOT), vasodilators, and neurotrophic drugs are optional treatments for ISSNHL, but their comparative efficacy is still uncertain. HBOT, as an optional treatment, has been used for ISSNHL since the 1960s.^4^ HBOT is believed to have complex positive effects on immunity, oxygen delivery, and hemodynamics, reducing hypoxia and edema, and enhancing the host's normal response to infection and ischemia.^5^ Since the cochlea's blood supply comes from the terminal artery, the labyrinthine artery, without collateral circulation, and cochlear hair cells have high metabolic activity, they are particularly susceptible to hypoxia or ischemic damage. Impaired cochlear perfusion in ISSNHL may lead to reduced oxygen concentration in the cochlear endolymph, increased blood viscosity, causing embolism and/or thrombosis, resulting in hearing loss.^6^, ^7^, ^8^ During HBOT, oxygen can diffuse through the terminal cochlear capillary network into the perilymph, increasing local oxygen pressure, improving blood oxygen supply and microcirculation, thereby improving the prognosis of ISSNHL.^9^^,^^10^

As an adjunctive treatment for ISSNHL, HBOT has been proposed in numerous studies, but there is still a lack of conclusive evidence to confirm its effectiveness. Some scholars even believe that HBOT does not significantly improve patient prognosis. In light of this, this study retrospectively analyzed the clinical data of 413 ISSNHL patients and used Propensity Score Matching (PSM) to evaluate the impact of HBOT combined with steroids as initial treatment on the prognosis of ISSNHL patients.

## Methods

Patients: This retrospective case-control study analyzed 413 ISSNHL inpatients (February 2021 ‒ June 2024). Data included: Demographics: Age, sex; Clinical features: Affected ear, tinnitus, vertigo, aural fullness; Comorbidities: Hypertension, diabetes; Disease course: Duration of illness; Audiologic metrics: Baseline/post-treatment hearing thresholds, hearing gain, hearing curve type, severity, and outcomes.

The inclusion criteria for patients were as follows: sudden sensorineural hearing loss occurring within 72 h, with hearing loss exceeding 30 dB in three consecutive frequencies; all cases were first-time unilateral occurrences; the time from onset to treatment was ≤14 days, and no prior treatment; age ≥18-years. Exclusion criteria included: bilateral sudden hearing loss; confirmed middle ear lesions, inner ear malformations, or retrocochlear space-occupying lesions upon examination; age <18-years, or women who were pregnant or breastfeeding; history of ear surgery, ototoxic drug use, or familial hereditary deafness-related diseases; conditions such as pneumothorax, acute sinusitis, retinal detachment, claustrophobia, or uncontrolled hypertension (systolic blood pressure ≥180 mm Hg); incomplete data.

### Hearing assessment & outcomes

All patients underwent comprehensive audiologic testing (pure-tone audiometry, immittance, OAEs, MRI).

Key measures: The Pure-Tone Average (PTA): Mean thresholds at 0.5, 1, 2, 4 kHz; Hearing levels were classified as normal (PTA ≤ 25 dB), mild hearing loss (PTA: 26–40 dB), moderate hearing loss (PTA: 41–60 dB), severe hearing loss (PTA: 61–80 dB), and profound hearing loss (PTA ≥ 81 dB). Additionally, ISSNHL cases were categorized into different types: low-frequency type (frequencies ≤ 1 kHz, with PTA ≥ 25 dB at 0.25 and 0.5 kHz), high-frequency type (frequencies ≥2 kHz, with PTA ≥ 25 dB at 4 and 8 kHz), flat type (all frequencies, with PTA ≤ 80 dB at 0.25, 0.5, 1, 2, 4, and 8 kHz), and total deafness type (PTA > 81 dB at all frequencies).^11^

Outcome classification (Siegel's criteria)^12^: Effective recovery: Complete (≤25 dB), partial (>15 dB gain, 26–45 dB), or slight (>15 dB gain, 46–75 dB), Ineffective: <15 dB improvement or >75 dB final threshold; Effective rate: (Complete + partial + slight recoveries) / total cases × 100%.

Treatment Methods: both groups received IV steroids: dexamethasone 10 mg/day (days 1–3), tapered to 5 mg/day (days 4–5); Intratympanic steroids: 0.5 mL dexamethasone every other day (5 total injections, starting day 2). HBOT commenced on the 3rd day of hospitalization, conducted at 2.5 absolute Atmospheres (ATA) for 120 min per session, once daily, for a total of 7 sessions. The HBOT protocol included three phases: compression (6–12 min to reach 2.5 ATA); maintenance (90 min at target pressure); decompression (5–15 min).

### Statistical methods

Continuous data are presented as mean ± standard deviation (x̄ ± s). Differences in baseline characteristics between the two groups were assessed using Pearson’s Chi-Square test or independent samples *t*-test. Non-parametric rank-sum tests were used to compare age, initial hearing level of the affected ear, post-treatment hearing level, hearing gain, degree of hearing loss, hearing curve type, and treatment efficacy between the two groups. A p-value <0.05 was considered statistically significant.

The PSM process was implemented using the “MatchIt” package in *R* software (version 4.2.2). Variables balanced between the two groups included gender, age, affected side, duration of illness, initial hearing level of the affected ear, tinnitus, vertigo, ear fullness, hypertension, diabetes, hearing curve type, and degree of hearing loss. Propensity scores were estimated using a logistic regression model, and 1:1 nearest neighbor matching was performed with a caliper value of 0.05. All analyses were conducted using *R* software (version 4.2.2). All p-values were two-sided, and the significance level (α) was set at 0.05.

## Results

There were no statistically significant differences in gender, age, and duration of illness between the two groups (p > 0.05). Additionally, the distributions of hypertension, diabetes, tinnitus, vertigo, and ear fullness were balanced and comparable between the two groups (p > 0.05). However, a statistically significant difference was observed in the affected side between the two groups (p < 0.05). The median initial PTA of the affected ear in the treatment group was 81 dB, compared to 71.5 dB in the control group, showing a statistically significant difference (p = 0.004). Significant differences were also observed in the degree of hearing loss and type of hearing loss between the two groups (p = 0.002 and p < 0.001). The effective rate in the treatment group was 43.9%, while it was 55.7% in the control group, with a statistically significant difference (p = 0.02) (Table 1).

**Table 1:** Comparison of characteristics between therapy and control groups before PSM.

Variables	Therapy group (n = 255)	Control group (n = 158)	p
Gender [Male n (%)]	127 (49.8)	71 (44.9)	0.336
Age [years, M (*P*_25_, *P*_75_)]	48 (37, 56)	50 (36.75, 61)	0.227
Affected side [Right n (%)]	136 (53.3)	63 (39.9)	0.008
Illness onset time [d, M (*P*_25_, *P*_75_)]	5 (3, 8)	4 (2, 7)	0.144
Initial hearing threshold [dB, M (*P*_25_, *P*_75_)]	81 (60, 103)	71.5 (44.75, 97)	0.004
Hearing gain [dB, M (*P*_25_, *P*_75_)]	12 (1, 33)	18 (4, 31.25)	0.210
Diabetes [n (%)]	47 (18.4)	38 (24.1)	0.170
Hypertension [n (%)]	55 (21.6)	47 (29.7)	0.061
Tinnitus [n (%)]	230 (90.2)	143 (90.5)	0.816
Vertigo [n (%)]	89 (34.9)	61 (38.6)	0.447
Ear fullness [n (%)]	48 (18.8)	29 (18.4)	0.905
Type of hearing loss [n (%)]			<0.001
Low frequency	11 (4.3)	25 (15.8)	
High frequency	12 (4.7)	7 (4.4)	
Flat-type frequency	100 (39.2)	70 (44.3)	
Total deafness	132 (51.8)	56 (35.4)	
Level of sudden hearing loss [n (%)]			0.002
Mild	22 (8.6)	26 (16.5)	
Moderate	47 (18.4)	36 (22.8)	
Severe	64 (25.1)	41 (28.9)	
Profound	122 (47.8)	55 (34.8)	
Outcome [n (%)]			0.02
Yes	112 (43.9)	88 (55.7)	
No	143 (56.1)	70 (44.3)	

Differences were considered statistically significant if p < 0.05.

To minimize baseline differences between the two groups, PSM analysis was performed. Using the nearest neighbor matching method, a 1:1 match was performed, resulting in 135 cases in each group. After matching, there were no significant statistical differences between the two groups in terms of gender, age, affected side, duration of illness, initial hearing level of the affected ear, hypertension, diabetes, tinnitus, vertigo, ear fullness, type of hearing loss, or degree of hearing loss (Table 2).

**Table 2:** Comparison of characteristics between therapy and control groups after PSM.

Variables	Therapy group (n = 135)	Control group (n = 135)	p
Gender [Male n (%)]	66 (48.9)	62 (45.9)	0.626
Age [years, M (*P*_25_, *P*_75_)]	51 (38, 59)	49 (38, 60)	0.802
Affected side [Right n (%)]	50 (37.0)	55 (40.7)	0.533
Illness onset time [d, M (*P*_25_, *P*_75_)]	4 (3, 7)	4 (2, 7)	0.477
Initial hearing threshold [dB, M (*P*_25_, *P*_75_)]	77 (57, 102)	75 (53, 100)	0.565
Diabetes [n (%)]	29 (21.5)	30 (22.2)	0.883
Hypertension [n (%)]	41 (30.4)	37 (27.4)	0.591
Tinnitus [n (%)]	120 (88.9)	122 (90.4)	0.690
Vertigo [n (%)]	48 (35.6)	50 (37.0)	0.800
Ear fullness [n (%)]	27 (20.0)	23 (17.0)	0.531
Type of hearing loss [n (%)]			0.371
Low frequency	11 (8.1)	15 (11.1)	
High frequency	9 (6.7)	4 (3.0)	
Flat-type frequency	52 (38.5)	62 (45.9)	
Total deafness	63 (46.7)	54 (40.0)	
Level of sudden hearing loss [n (%)]			0.676
Mild	17 (12.6)	17 (12.6)	
Moderate	28 (20.7)	29 (21.5)	
Severe	33 (24.4)	37 (27.4)	
Profound	57 (42.2)	52 (38.5)	

Differences were considered statistically significant if p < 0.05.

After propensity score matching, the median hearing gain in the treatment group was 13 dB, while it was 19 dB in the control group, showing no statistically significant difference (p = 0.131). The effective rate was 47.4% in the treatment group and 55.6% in the control group, with no statistically significant difference (p = 0.180) (Table 3).

**Table 3:** Hearing outcome in the therapy and control group after PSM.

Variables	Therapy group (n = 135)	Control group (n = 135)	p
Hearing gain [dB, M (*P*_25_, *P*_75_)]	13 (2, 30)	19 (4, 33)	0.131
Effectiveness			0.180
Yes	64 (47.4)	75 (55.6)	
No	71 (52.6)	60 (44.4)	

After PSM, patients with severe and profound hearing loss were selected, including 90 in the Therapy group and 89 in the control group. Post-treatment hearing levels improved significantly in both groups, with notable differences compared to pre-treatment levels. However, there was no significant difference in hearing gain between the two groups (p = 0.559). The effective rate was 47.8% in the therapy group and 48.3% in the control group, with no statistically significant difference between the two (p = 0.943) (Table 4).

**Table 4:** PTA values before and after the treatment, hearing gains and recovery rates with severe and profound hearing loss after PSM.

Groups	Pre-treatment PTA dB, M (P_25_, P_75_)	Post-treatment PTA dB, M (P_25_, P_75_)	p-value	Hearing gain dB, M (P_25_, P_75_)	p-value	Outcome		p-value
						Yes (%)	No (%)	
Therapy group (n = 90)	95 (75, 107)	73 (41.5, 96.5)	<0.001	16.5 (2.75, 39.5)		47.8	52.2	
Control group (n = 89)	91 (74.5, 113)	66 (37.5, 92.5)	<0.001	21 (2.5, 43.5)	0.559	48.3	51.7	0.943

PTA, Pure Tone Average, HBOT, Hyperbaric Oxygen Treatment.

Differences were considered statistically significant if p < 0.05.

## Discussion

ISSNHL is an acute hearing loss of unknown etiology that can occur at any age. Its incidence increases with age.^13^ This condition is often accompanied by symptoms such as hearing loss, tinnitus, and vertigo, and may trigger anxiety and fear, significantly impacting patients' quality of life, work efficiency, and social activities.^14^

The prognosis of ISSNHL is multifactorial, with reported cure rates demonstrating considerable variability. Despite aggressive therapeutic interventions, over 50% of patients fail to achieve complete hearing recovery.^15^^,^^16^ In recent years, HBOT has emerged as a promising adjunctive treatment, particularly when combined with corticosteroid therapy, to enhance clinical outcomes in ISSNHL patients. The 2019 American Clinical Practice Guidelines for Sudden Hearing Loss endorse HBOT as both a potential initial treatment option within the first two weeks of symptom onset and a salvage therapy for non-responders to initial pharmacological treatment within one month.^1^ Correspondingly, the Chinese Guidelines for the Treatment of Sudden Deafness recommend HBOT as a viable therapeutic adjunct, frequently administered in conjunction with conventional treatment modalities.^11^

In our study, the pre-PSM treatment efficacy analysis revealed a significantly lower effective rate in the Therapy group (43.9%) compared to the control group (55.7%), with a statistically significant difference (p = 0.02), suggesting that HBOT may enhance therapeutic outcomes in ISSNHL patients. However, substantial baseline disparities were observed between the groups regarding several prognostic factors: affected side, initial hearing thresholds, degree of hearing loss, and type of hearing loss. Notably, pretreatment assessment demonstrated significant intergroup differences in initial hearing thresholds. The Therapy group exhibited a median initial PTA of 81 dB in the affected ear, significantly higher than the control group's 71.5 dB (p = 0.004). Furthermore, significant differences were observed in the type of hearing loss (p < 0.001): therapy group had a lower proportion of low-frequency hearing loss (4.3% vs. 15.8%) but a higher proportion of total deafness (51.8% vs. 35.4%) compared to the control group. Additionally, the severity distribution of hearing loss differed significantly between groups, with the therapy group showing a higher prevalence of profound hearing loss (47.8% vs. 34.8%, p = 0.002).

Studies have shown that the initial severity of hearing loss is an important factor affecting prognosis, with patients experiencing severe to profound hearing loss having lower recovery rates.^17^, ^18^, ^19^ Other studies have revealed that the initial hearing threshold and the hearing curve are also key indicators for predicting hearing recovery outcomes. Specifically, patients with flat-type or total deafness-type hearing loss tend to have poorer prognoses.^20^, ^21^, ^22^ Additionally, research indicates that the prognosis of low-frequency ISSNHL patients is significantly better than that of high-frequency patients.^23^ Therefore, the significant imbalance in covariates between the treatment and control groups may lead to biased treatment outcomes, potentially affecting the true efficacy of HBOT as an adjunctive therapy.

PSM is a practical and innovative statistical method for analyzing non-randomized controlled data, effectively balancing covariates between groups. Therefore, this study employed PSM on the basis of a case-control design to enhance the balance of covariates in non-randomized grouped data, ensuring comparability between the treatment and control groups. In the study sample, significant differences were observed between the two groups in variables such as initial hearing level of the affected ear, type of hearing loss, and degree of hearing loss. Gender is an important demographic characteristic, while tinnitus and ear fullness are common symptoms. Additionally, although factors such as duration of illness, age, and comorbidities showed no statistical differences in this study, they are recognized as important factors influencing the prognosis of ISSNHL.^24^, ^25^, ^26^, ^27^ Therefore, these factors were also included as covariates.

After PSM, 135 cases were included in each of the treatment and control groups. Post-PSM, there were no statistically significant differences between the therapy and control groups in terms of gender, age, affected side, duration of illness, initial hearing level of the affected ear, hypertension, diabetes, tinnitus, vertigo, ear fullness, type of hearing loss, or degree of hearing loss. After PSM, the effective rate in the therapy group was 47.4%, compared to 55.6% in the control group, with no statistically significant difference. These results suggest that the addition of HBOT to IVS and IT as an initial therapy for ISSNHL did not further improve hearing outcomes in ISSNHL patients.

Several studies have reached similar conclusions. One study compared steroid therapy alone with steroid therapy combined with HBOT and found no significant differences in hearing gain or recovery rates between the two groups. The study also concluded that adding HBOT to intravenous and intratympanic corticosteroid treatment did not affect hearing recovery, regardless of the degree of hearing loss.^28^ Another study reported response rates of 50.8% for oral corticosteroids alone and 61.5% for oral corticosteroids combined with HBOT, but the difference was not statistically significant.^29^ Additional research has also indicated that HBOT, as an adjunctive treatment for ISSNHL, did not result in statistically significant differences in hearing outcomes.^30^^,^^31^

In our study, all included patients were receiving treatment for the first time after the onset of symptoms, effectively eliminating bias from prior treatments. However, ISSNHL has a certain degree of spontaneous recovery, with spontaneous recovery rates ranging from approximately 1/3 to 2/3, and such recovery typically occurs within the first two weeks after onset.^1^ Since the patients in our study received HBOT within two weeks of symptom onset, it is possible that the benefits of HBOT could not be accurately assessed.

Additionally, Liu et al. suggested that HBOT as an adjunct to pharmacological treatment could benefit patients with profound hearing loss.^32^ Ajduk et al. also reported that patients with severe and profound hearing loss showed significant hearing improvement after receiving a combination of steroids and HBOT.^33^ Therefore, after PSM, we specifically analyzed patients with severe and profound hearing loss. Although both groups exhibited significant hearing improvement after treatment, there was no statistically significant difference in the overall effective rate between the two groups (p = 0.943). Our findings indicate that adding HBOT as an initial adjunctive treatment to systemic and intratympanic steroids did not further improve hearing outcomes in patients with severe and profound hearing loss. This conclusion aligns with other studies, which have shown that HBOT as an adjunct to pharmacological treatment does not improve the PTA in patients with severe to profound ISSNHL.^28^^,^^30^

However, some studies have demonstrated that combining HBOT with corticosteroids has significant benefits as an initial treatment for ISSNHL patients.^34^, ^35^, ^36^, ^37^ Compared to corticosteroid treatment alone, the combination therapy yields better outcomes.^38^ Wang et al. found that patients receiving HBOT had significantly higher average PTA thresholds than those who did not receive HBOT, with the difference being statistically significant. Furthermore, the study emphasized the importance of initiating HBOT early for improving hearing outcomes in ISSNHL patients.^39^

Thus, the efficacy of HBOT as an initial treatment for ISSNHL remains controversial. Future large-scale, multicenter, prospective studies are needed to further investigate this issue.

This study has several limitations. First, as a case-control study, selection bias and recall bias were inevitable. However, the use of PSM balanced baseline characteristics between the two groups and controlled for confounding factors, effectively reducing recall bias. Additionally, this study did not comprehensively assess the long-term prognosis of patients. Future research could adopt a prospective design, expand the sample size, and incorporate multicenter data to further validate and extend the findings of this study.

## Conclusions

In summary, the application of PSM effectively improved the balance of covariates between non-randomized groups. After PSM adjustment, our results showed no significant difference in the overall effective rate between the therapy and the control group. Similarly, no statistically significant difference was observed in the overall effective rate among patients with severe and profound hearing loss. In conclusion, we found that adding HBOT as an initial treatment modality for ISSNHL, when combined with intravenous and intratympanic steroid therapy, does not provide additional improvement in outcomes for patients. However, the inherent selection bias in retrospective studies may influence the results, necessitating further confirmation through prospective research.

## ORCID ID

Weihong Hong: 0009-0009-9981-6598

Lipeng Huang: 0009-0002-5079-8893

Yongjun Hong: 0009-0004-3164-7584

## Funding

This work was supported by the Fujian Health and Youth Scientific Research Program, grant number (2024QNB016).

## Data availability statement

The authors declare that all data are available in repository.

## Declaration of competing interest

The authors declare no conflicts of interest.
